# AlergiaPT: A Portuguese media campaign to inspire people with allergies to make a positive change in their life

**DOI:** 10.1097/j.pbj.0000000000000169

**Published:** 2022-02-08

**Authors:** André Moreira, Francisca de Castro Mendes, Tiago Rama, Diogo Mota, Diana Silva, Inês Pádua, Cristina Abreu, Maria João Vasconcelos, Mariana Farraia, Inês Paciência, João Rufo, Renata Barros, Patrícia Padrão, Pedro Moreira, Diana Seabra, Henrique Barros

**Affiliations:** a EPIUnit, Institute of Public Health, University of Porto, Portugal & Laboratory for Integrative and Translational Research in Population Health (ITR), Porto, Portugal; b Serviço de Imunoalergologia, Centro Hospitalar Universitário São João, Porto, Portugal; c Basic and Clinical Immunology Unit, Department of Pathology, Faculty of Medicine, University of Porto, Porto, Portugal; d Faculty of Nutrition and Food Sciences, Porto, Portugal.

**Keywords:** allergy, anaphylaxis, asthma, public health

## Abstract

Allergic diseases comprise a significant cause of morbidity worldwide and a substantial burden on the health and medical systems of both developed and emerging economies. Although highly prevalent, relatively severe, and largely impactful on the quality of life of patients, allergic diseases are commonly trivialized. Increasing awareness of the relevance of allergic diseases as a major public health problem might lead to an improved acknowledgment by governments and health authorities. Based on the positive impact that media campaigns might have on health-related behaviors, as well as the large use of social media by different types of users, social media might be used as a powerful tool for spreading awareness and education even more effective than traditional face-to-face communication. Therefore, we aimed to develop a social media-based communication program, the AlergiaPT, reaching all stakeholders, to increase the awareness of allergic diseases tackling the causes, prevention, control, and economic impact. The AlergiaPT will provide user-generated and interactive content toward engagement, include both long-form and short-form video productions toward education, as well as stories and time-sensitive content toward empowerment. It will be targeted to all populations, engaging different stakeholders. Contents will address the 5 campaign goals: i) allergy health is promoted; ii) tolerance is actively reinforced, and avoidance reduced; iii) treatment control and guided self-management of patients of asthma, rhinitis, food allergy, and atopic eczema are strengthened; iv) recognition and treatment of severe allergy and anaphylaxis are improved, and v) indoor air quality is promoted. Engagement on the campaign will be promoted through stepwise educational takeaways meetings using different social media, and targeting all audience groups, by promoting the organization of resources for common goals and the involvement of social media to improve public awareness. The impact of AlergiaPT will be assessed through google analytics.

## Why do we need an allergy health ecampaign?

Allergic diseases comprise a significant cause of morbidity worldwide and a substantial burden on the health and medical systems of both developed and emerging economies. Allergies have become the most frequent chronic disease in the European Union affecting at least 150 million Europeans and nearly 80% of families.^1^ Allergy covers a wide spectrum of diseases, many of them being very frequent, such as allergic rhinitis, allergic asthma, atopic dermatitis, urticaria, and life-threatening food, drug, and stinging insect venom reactions. According to recent studies, their prevalence is increasing globally.^2^ Six hundred thousand Portuguese people have asthma, with allergy being the cause in 80% of cases.^3^ Although its high prevalence, relative severity, and a huge impact on quality of life, allergic diseases are habitually trivialized. This is perceived through all segments of society, beginning at the medical schools where allergies have very little room in the curricula, to the media, and in politics as well as in research funding, where allergies are almost always taken less seriously than other medical conditions with similar potentially fatal results.

Since allergic conditions are often perceived by patients as trivial conditions, it is not surprising that more than half of individuals with allergic rhinitis have not seen a medical professional in the previous year and almost one-third preferred nonprescription medication because they considered it not necessary to visit a doctor.^4^ Yet, for an important number of patients, with more severe or persistent diseases, symptoms are inadequately controlled by pharmacological treatment. The number of untreated or incorrectly treated patients, who are consequently partly or fully symptomatic, in all studies is approximately 90%.^5^ In these cases, the bothersome nature of symptoms can severely affect daily activities such as the ability to work, examination performance, quality of life, and psychosocial well-being. Moreover, allergies can be deemed a significant public health problem, which often requires the use of emergency care, sometimes including hospital admission, and are responsible for a high number of missed school and/or workdays. Overall, allergy-related costs may be considered very high. In Europe, indirect costs per under-treated allergic patient are €2405 per year.^5^ In 2010, 2% of the annual health expenditure of Portugal, that is, about 360 million Euros, was invested only in asthma.^6^ On the other hand, effective therapy for allergic diseases is available for a relatively low cost (€125) per patient per year, that is, 5% of the costs of an insufficiently treated disease. Therefore, there is a significant margin for cost reduction and for a positive impact on health indicators.

Increasing awareness on the relevance of allergic diseases as a major public health problem might lead to an improved acknowledgment by governments and health authorities. The Global Asthma Network recommended that, in all countries, national asthma strategies should be developed together with national action plans to improve early asthma detection, increase asthma management, and reduce costs.^7^ Probably the world's most successful national asthma program ever has been the Finnish Asthma Programme 1994–2004, whose experience was fundamental in designing and implementing the world-leading program on allergy prevention—the Finnish Allergy Program 2008–2018.^8^ The Finnish Allergy Programme 2008–2018 was introduced to test new thinking in practice, and regarding the improvement in allergy health, including asthma-related health. Nonetheless, in the context of allergies prevention, strategies and recommendations have typically been exaggerated and restrictive based on relatively poor scientific evidence, while the Finnish Allergy Program has changed the paradigm. A public health program was implemented, and an avoidance strategy was replaced with a tolerance strategy. These strategies were developed by health authorities and governments and evaluated, reported, and published.^8^

Media campaigns may produce positive or prevent negative changes in health-related behaviors across large populations. Such campaigns have most particularly been aimed at tobacco use and heart-disease prevention, but have also addressed alcohol and illicit drug use, cancer screening and prevention, sex-related behaviors, and many other health-related issues. Typical campaigns have placed messages in media that reach large audiences, most frequently via television or radio, but also outdoor media, such as billboards and posters, and print media, such as magazines and newspapers. In the past, exposure to such messages was generally passive, resulting from an incidental effect of routine use of media. Currently, social media is a space to connect people with common interests. With the continuous expansion of technology, it is emerging as a powerful tool in spreading awareness and education of various socially relevant concepts, and conducting activities, online courses, and classes more effectively than face-to-face direct communication. Websites such as Facebook, Twitter, TikTok, and Instagram, collectively termed social media, facilitate many-to-many communication instead of the traditional personal one-to-one communication and one-to-many communication. Social media, over 10 years, have actively worked as a catalyst in changing the dynamics of communication and work culture. Hence, we aimed to develop a social media-based communication program, reaching all stakeholders, to increase the awareness of allergic diseases tackling the causes, prevention, control, and economic impact.

## Which goals (engage, educate, and empower) and target audiences (diversity, equity, and inclusion)?

Based on the key messages of the Finnish Allergy Programme 2008–2018,^8–10^ the Portuguese National Allergy Media Campaign (AlergiaPT) focuses on i) endorsing allergy health, ii) strengthening tolerance, iii) adopting a new attitude to allergy by avoiding allergens only if mandatory, iv) raising awareness to/recognizing a severe allergy, v) preventing exacerbations, and vi) improving indoor air quality through promotion of cigarette smoke-free environments. Briefly, AlergiaPT will provide user-generated and interactive content toward engagement, including both long-form and short-form video productions for education, as well as stories and time-sensitive content toward empowerment.

The AlergiaPT will be targeted to all populations, engaging different stakeholders, including healthcare staff, pharmacists, schools’ staff and educators, scientific societies, patient associations, and laypeople, namely patients with allergies and their families.

## Contents and strategies for development and implementation: networking will be the key to success!

The use of social networks has grown among people of all ages, allowing health communications to be more dynamic and reach more communities. In addition to its webpage, the AlergiaPT will have profiles on Facebook, Instagram, TikTok, and Twitter to distribute its contents. Through this communication strategy, we intend to introduce new evidence-based knowledge, engage people on online events that may target people directly in their communities, and provide education and publicity for change through counseling.

**Figure 1:**
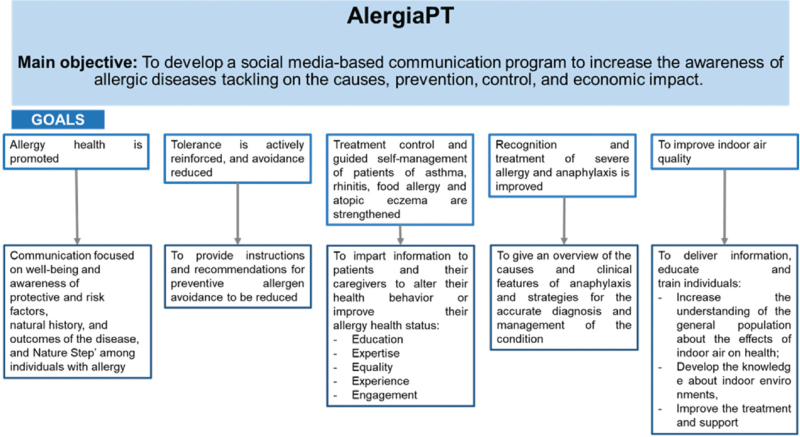
The 5 campaign goals of AlergiaPT, which are targeted to all populations, engaging different stakeholders.

Contents will address the 5 campaign goals (Fig. 1):

### Goal 1: Allergy health is promoted

Communication will focus on well-being and awareness of protective and risk factors, natural history, and outcomes of the disease, as well as the “Nature Step” among individuals with allergies.

Allergy health reports to physical, psychological, and social well-being irrespective of allergy.^8^ An individual can be healthy and functional, although allergic. In this case, allergy is recognized as an individual feature rather than an illness, when the condition is minor and relative. Allergy health is endorsed through a balanced diet, physical activity, and a close connection with the natural environment. This will promote general health and immune balance. Moreover, activities fulfilling this goal will be compliant and endorse the Nature Step in trying to reset the connection between individuals and the natural environment.^11^ Planetary health is defined as the health of human civilization and the state of the natural systems on which it depends.^8,11–15^ Planetary health and allergy health are interconnected, and both need to be considered by individuals and governments while addressing several UN Sustainable Development Goals.

### Goal 2: Tolerance is actively reinforced and avoidance reduced

The previous paradigm promoted avoidance of allergens as a means to prevent allergic diseases. Although relevant in specific situations, there is increasing evidence showing that in addition to not being an adequate strategy to reverse the incidence and prevalence of allergic diseases, allergen avoidance might also have long-term consequences.^16^ In addition, except for specific cases such as anaphylaxis, there are no long-term benefits of allergen avoidance. Moreover, the accumulating evidence also suggests that the prevention of allergic diseases might be achieved through the development of allergen tolerance. Therefore, tolerance to allergens must be improved.^10^ The AlergiaPT education campaign will promote the increase of allergens tolerance by providing instructions and recommendations for preventive allergen avoidance to be reduced.

### Goal 3: Treatment control and guided self-management of patients with asthma, rhinitis, food allergy, and atopic eczema are strengthened

Patient empowerment is the process by which AlergiaPT health professionals and others impart information to patients and their caregivers that will alter their health behavior or improve their allergy health status. Within this goal there are 5 key crucial aspects: i) Education: patients can make informed decisions about their health if they can access all the relevant information, in an easily understandable format; ii) Expertise: patients self-manage their condition every day so they have unique expertise on healthcare which needs to be supported; iii) Equality: patients need support to become equal partners with health professionals in the management of their condition; iv) Experience: individual patients work with patient organizations to represent them and channel their experience and collective voice; and v) Engagement: patients need to be involved in designing more effective healthcare for all, and in research to deliver new and better treatments and services.

### Goal 4: Recognition and treatment of severe allergy and anaphylaxis are improved

Prompt recognition and management of anaphylaxis are imperative.^17–19^ However, the condition is often under-recognized and treated inadequately. Diagnosis and management are challenging since reactions are often immediate and unexpected. The campaign will provide an overview of the causes and clinical features of anaphylaxis, as well as strategies for the accurate diagnosis and management of the condition.

### Goal 5: To improve indoor air quality

Nowadays, the impact of outdoor and indoor air quality in allergic and respiratory diseases prevalence is widely recognized.^20–22^ Although the general population is aware of the importance of outdoor air quality to their health status, they are less informed about the adverse health effects that are associated with indoor air pollution.^23^ Considering the complexity of issues related to indoor air quality, there is a need to improve, simultaneously, the management and prevention of indoor air pollutants.^24^ Moreover, exposure to tobacco smoke, either directly and indirectly, should be avoided considering its effect on the severity and development of allergic conditions, including asthma. In this context, the AlergiaPT will be committed to provide information, educate, and train individuals regarding the importance of indoor air quality. Specifically, it will increase the understanding of the general population about the effects of indoor air quality on health as well as develop the knowledge about indoor environments, while improving the treatment and support to individuals.

## Creating an engaging allergy health promotion campaign on social media

**Figure 2:**
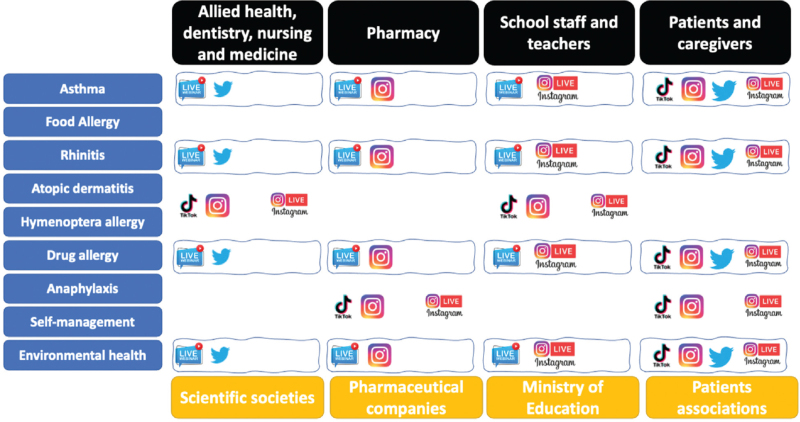
AlergiaPT content marketing matrix—framework targeting all populations, engaging different stakeholders with interchangeable dynamic messages contents.

AlergiaPT will take advantage of stepwise online educational meetings targeting all audience groups (Fig. 2). Particularly, considering that people with a mild or suspected allergy might not be in contact with a specialist, he/she tends to search for answers and information online, including social media or websites [6]. Therefore, AlergiaPT will be committed to creating online information arenas to combat unjustified fear and avoidance of exposure. For this lay audience, we intend to change the attitude toward the prevention of allergic diseases. Different formats for these short, interactive, live-streamed takeaway meetings will be designed according to the target audience and message content. AlergiaPT will provide online-based eLearning modules for patients and their caregivers for guided self-management to manage exacerbations/attacks; and online-based eLearning networking modules with specialists, general practitioners, nurses, and pharmacists focusing on asthma and allergy health in diagnostics to improve early detection and in treatment to improve the effective use of drugs. The contents of the proposed modules are defined according to the objectives of the program. In addition, the themes/topics included in each module will consider the needs/interests of the participants. Although these meetings are held in real-time, they will be available on social media AlergiaPT pages so that they can be viewed anywhere and on-demand.

AlergiaPT will develop a mobile-based interactive learning environment taking advantage of social media profiles on different platforms having the opportunity to reach more people. On the main website, the AlergiaPT team will regularly share photos/videos, links, texts, games, polls, or questionnaires in the context of allergy to engage the general population. These posts/publications will include informative content, providing information about allergic diseases as a public health problem, associated risk/protective factors (eg, behavioral/lifestyle), and the potential consequences and benefits achieved from the adoption of some behaviors. They will also instill the “call to action” messages to encourage users to become actively involved in the campaign (ie, sharing programs or events), as well as instructive sharing, where instructions/guidelines on certain behaviors will be presented (ie, management of adrenaline auto-injectors). To promote positive changes and increase campaign outreach, social media publications will share messages that aim to generate positive feelings for the campaign. On the other hand, “less positive” content and contexts will also be shared to draw attention to possible problems and increase sensitivity/knowledge about allergic diseases. In addition, the AlergiaPT campaign will disseminate real testimonies that will share their experience and day-to-day life as patients, family, or healthcare professionals. The support and engagement of celebrities and media influencers who will advertise and campaign. This strategy aims to reach a greater number of users and for them to actively adhere and participate in the campaign while increasing awareness about allergies since celebrities can influence their followers. In addition, experts, such as physicians, researchers, and other professionals, will also be invited to share and be committed to the campaign.

For other stakeholders, such as school communities, we will promote competitions and contests between schools and among students who will have the collaboration from schools’ staff and educators, as well as families. These activities aim to involve these communities directly in the campaign by sharing photos, videos (ie, dancing and singing), stories with messages in the allergy context while spreading the campaign to more users. Users will have to tag AlergiaPT's social media pages, and winners will be selected based on engagement (ie, likes and shares).

### Promote the organization of resources for common goals

The AlergiaPT team will be working with scientific societies, and patient associations, to produce educational material for professionals, the general population, and patients and their families. These educational materials together with content from the online takeaway meetings will be available at the AlergiaPT website if the speakers agree. In addition, the AlergiaPT website will include all the information corresponding to the campaign, including information about the team, goals, social media, events/educational takeaways meetings (eg, dates, times, and content details), publications, contests, and competitions (eg, instructions, participants, deadlines, and winners) and other information (eg, curiosities and key messages). Guidelines for patients and parents/caregivers of allergic children will also be available. A question-and-answer section will also be available to increase the interaction between all stakeholders.

Pharmaceuticals and industries will also be considered interest groups and stakeholders. Sponsorships and partnerships will be established so that participants and winners of contests and competitions receive prizes, which may include vouchers, offers, discounts, among others.

## Process evaluation of AlergiaPT

Google Analytics will be used to access user data over the first year of use and evaluate and track the impact of the AlergiaPT campaign. We will be able to improve the understanding of the audience that is following and visiting the campaign and create custom segments to identify specific subgroups. In addition, Google Analytics will provide information about the geolocation and behavior of the AlergiaPT audience (eg., new users, frequency, and engagement). Nonetheless, the data provided from Google Analytics does not contain any personally identifiable information and is presented in an aggregated form, making it possible to use them without ethical concerns. Information provided will be summarized in a real-time, interactive dashboard, which will be accessed by logging in.

The assessment will include several parameters, such as awareness, traffic to the campaign website, engagement, and post-engagement, and account mentions. Specifically, awareness will be evaluated based on metrics measuring volume, reach, exposure, and amplification, while traffic to the campaign website will consider URL shares, clicks, and conversions. In addition, engagement and post-engagement rate will be measured through likes, comments, shares and clicks, and the number of engagements divided by impressions or reach, whereas account mentions will consider like and tag (@) mentions that are not part of a reply or tagging in a Facebook/Instagram/Twitter publication within 24 hours without prompting.

## AlergiaPT: only fighting ignorance guarantees the virtue of tolerance

AlergiaPT intends to inspire Portuguese people with allergies to adopt a more positive attitude toward life, through a social media-based campaign. Officially launched on 2021 World Asthma Day, the project aims to promote allergy tolerance and foster a new attitude toward allergies among people, prevent the exacerbation of severe allergies, and alert to the importance of improving air quality. To achieve these goals, a communication strategy based on social media, with content directed to the various target audiences of the project such as the general population, health professionals, pharmacists, schools, scientific societies, and patient associations will be produced by the project team. In conclusion, AlergiaPT will commit to the enchanting immune global concept according to which only fighting ignorance guarantees the virtue of tolerance.

## Acknowledgments

The TIPH (This is Public Health) Global Grant Program is a global funding program that rewards initiatives related to the dissemination and promotion of the importance of public health. Created by the Association of Schools and Programs of Public Health (ASPPH), it has established partnerships with associations of schools of public health from around the world, including the Association of Schools of Public Health in the European Region (ASPHER). In 2021, 20 international projects were awarded, one of which was AlergiaPT. More information about the program and the winning projects is available here: https://www.aspph.org/tiph-global-grant-program-awardees-announced/

## Conflicts of interest

The authors have no conflicts of interest to disclose.

## References

[1] Sanchez-Borges M Martin BL Muraro AM The importance of allergic disease in public health: an iCAALL statement. World Allergy Organ J. 2018;11 (1):8. 29743965 10.1186/s40413-018-0187-2 PMC5921992

[2] Asher MI Garcia-Marcos L Pearce NE Strachan DP. Trends in worldwide asthma prevalence. Eur Respir J. 2020;56 (6):10.1183/13993003.02094-2020 32972987

[3] Barros R Moreira P Padrao P Obesity increases the prevalence and the incidence of asthma and worsens asthma severity. Clin Nutr. 2017;36 (4):1068–1074. 27448950 10.1016/j.clnu.2016.06.023

[4] Maurer M Zuberbier T. Undertreatment of rhinitis symptoms in Europe: findings from a cross-sectional questionnaire survey. Allergy. 2007;62 (9):1057–1063. 17581263 10.1111/j.1398-9995.2007.01367.x

[5] Zuberbier T Lotvall J Simoens S Subramanian SV Church MK. Economic burden of inadequate management of allergic diseases in the European Union: a GA(2) LEN review. Allergy. 2014;69 (10):1275–1279. 24965386 10.1111/all.12470

[6] Barbosa JP Ferreira-Magalhaes M Sa-Sousa A Azevedo LF Fonseca JA. Cost of asthma in Portuguese adults: a population-based, cost-of-illness study. Rev Port Pneumol (2006). 2017;23 (6):323–330. 28807558 10.1016/j.rppnen.2017.07.003

[7] “National and regional asthma programmes in Europe.” Olof Selroos, Maciej Kupczyk, Piotr Kuna, Piotr Lacwik, Jean Bousquet, David Brennan, Susanna Palkonen, Javier Contreras, Mark FitzGerald, Gunilla Hedlin, Sebastian L. Johnston, Renaud Louis, Leanne Metcalf, Samantha Walker, Antonio Moreno-Galdo, Nikolaos G. Papadopoulos, Jose Rosado-Pinto, Pippa Powell and Tari Haahtela. Eur Respir Rev 2015; 24: 474–483. Eur Respir Rev. 2019; 28(154). 10.1183/16000617.5081-2014 PMC9489041 31871128

[8] Haahtela T Valovirta E Bousquet J Mäkelä M. Allergy Programme Steering Group. The Finnish Allergy Programme 2008–2018 works. Eur Respir J. 2017;49 (6):10.1183/13993003.00470-2017 28642312

[9] von Hertzen LC Savolainen J Hannuksela M Scientific rationale for the Finnish Allergy Programme 2008–2018: emphasis on prevention and endorsing tolerance. Allergy. 2009;64 (5):678–701. 19383025 10.1111/j.1398-9995.2009.02024.x

[10] Haahtela T von Hertzen L Mäkelä M Hannuksela M. Allergy Programme Working Group. Finnish Allergy Programme 2008–2018 – time to act and change the course. Allergy. 2008;63 (6):634–645. 18445181 10.1111/j.1398-9995.2008.01712.x

[11] Haahtela T von Hertzen L Anto JM Helsinki by nature: the nature step to respiratory health. Clin Transl Allergy. 2019;9:57. 31695865 10.1186/s13601-019-0295-2 PMC6822361

[12] Ruokolainen L Fyhrquist N Haahtela T. The rich and the poor: environmental biodiversity protecting from allergy. Curr Opin Allergy Clin Immunol. 2016;16 (5):421–426. 27490122 10.1097/ACI.0000000000000304

[13] Haahtela T Alenius H Lehtimaki J Immunological resilience and biodiversity for prevention of allergic diseases and asthma. Allergy. 2021. 10.1111/all.14895 33959980

[14] Haahtela T. A biodiversity hypothesis. Allergy. 2019;74 (8):1445–1456. 30835837 10.1111/all.13763

[15] Haahtela T. Why medical community should take biodiversity loss seriously? Porto Biomed J. 2017;2 (1):4–5. 32258576 10.1016/j.pbj.2016.10.007 PMC6806989

[16] Boolchandani H Horwitz R Soffer G. An integrative medicine review of primary prevention of allergy in paediatrics. Complement Ther Med. 2021;58:102695. 33636296 10.1016/j.ctim.2021.102695

[17] de Silva D Singh C Muraro A Diagnosing, managing and preventing anaphylaxis: systematic review. Allergy. 2021;76 (5):1493–1506. 32880997 10.1111/all.14580

[18] Muraro A Worm M Alviani C EAACI guideline: anaphylaxis (2021 update). Allergy. 2021. 10.1111/all.15032 34343358

[19] Kraft M Dolle-Bierke S Turner PJ EAACI taskforce clinical epidemiology of anaphylaxis: experts’ perspective on the use of adrenaline autoinjectors in Europe. Clin Transl Allergy. 2020;10:12. 32426107 10.1186/s13601-020-00317-y PMC7216364

[20] Garcia E Berhane KT Islam T Association of changes in air quality with incident asthma in children in California, 1993–2014. JAMA. 2019;321 (19):1906–1915. 31112259 10.1001/jama.2019.5357 PMC6537847

[21] Thurston GD Rice MB. Air pollution exposure and asthma incidence in children: demonstrating the value of air quality standards. JAMA. 2019;321 (19):1875–1877. 31112243 10.1001/jama.2019.5343

[22] Poole JA Barnes CS Demain JG Impact of weather and climate change with indoor and outdoor air quality in asthma: A Work Group Report of the AAAAI Environmental Exposure and Respiratory Health Committee. J Allergy Clin Immunol. 2019;143 (5):1702–1710. 30826366 10.1016/j.jaci.2019.02.018 PMC10907958

[23] Seguel JM Merrill R Seguel D Campagna AC. Indoor air quality. Am J Lifestyle Med. 2017;11 (4):284–295. 30202344 10.1177/1559827616653343 PMC6125109

[24] Lampi J Hyvarinen A Erhola M Healthy people in healthy premises: the Finnish Indoor Air and Health Programme 2018–2028. Clin Transl Allergy. 2020;10:4. 31969979 10.1186/s13601-020-0308-1 PMC6966831

